# Maternal anemia in helminth-infected pregnant women: Dose–response with hookworm intensity and other risk factors in a Ugandan cross-sectional study

**DOI:** 10.1371/journal.pntd.0014277

**Published:** 2026-04-30

**Authors:** Geoffrey Akena, Marie Pascaline Sabine Ishimwe, Theodore Nteziyaremye, Maxwell Okello, Ahmed Kiswezi Kazigo, Theoneste Hakizimana

**Affiliations:** 1 Department of Obstetrics and Gynecology, Kampala International University, Ishaka, Uganda; 2 Department of Pediatrics and Child Health, Kampala International University, Ishaka, Uganda; 3 Department of Surgery, Kampala International University, Ishaka, Uganda; 4 Department of sciences, University of Rwanda, Kigali, Rwanda; University of Oxford, UNITED KINGDOM OF GREAT BRITAIN AND NORTHERN IRELAND

## Abstract

Anemia remains a significant global public health challenge, affecting approximately 25% of the world’s population and disproportionately impacting pregnant women, with an estimated 40% prevalence worldwide. Intestinal helminth infections exacerbate this burden, with anemia prevalence reported as high as 55.6% among infected pregnant women compared to 16.4% in those uninfected. This study aimed to determine the burden of anemia and its determinants among pregnant women with helminthiasis attending antenatal care at tertiary Hospitals in western Uganda. Methods: A hospital-based cross-sectional study was conducted from 1^st^ November 2022–1^st^ March 2023, enrolling 420 pregnant women diagnosed with helminthiasis attending antenatal clinics. Data were collected via interviewer-administered questionnaires and laboratory records. Descriptive statistics and multivariable logistic regression analyses were performed using IBM SPSS version 23 to identify factors independently associated with anemia. Results: The prevalence of anemia was 26.2% (95% CI: 22.2–30.6), with 39.1% (95% CI: 30.5–48.4) mild, 35.5% (95% CI: 27.1–44.7) moderate, and 25.5% (95% CI: 18.2–34.3) severe cases. Multivariate analysis showed that moderate and heavy hookworm intensities increased anemia risk nearly two-fold (aOR = 1.90, 95% CI: 1.16–3.54; p = 0.028) and 2.4-fold (aOR = 2.40, 95% CI: 1.06–5.41; p = 0.041), respectively. Protective factors included being a student (aOR = 0.17, 95% CI: 0.05–0.63; p = 0.008), deworming ≥3 months prior (aOR = 0.11, 95% CI: 0.01–0.88; p = 0.038), using borehole water (aOR = 0.60, 95% CI: 0.36–1.00; p = 0.048), and gestational age 14–27 weeks (aOR = 0.57, 95% CI: 0.35–0.92; p = 0.020). Conclusions: Maternal anemia showed a clear dose–response with hookworm intensity, with risk increasing from moderate to heavy infection. Delayed deworming and unsafe water increased odds, whereas being a student and second-trimester gestation were protective. Timely anthelminthic treatment, improved water access, and strengthened antenatal care are critical in endemic settings.

## Author summary

Anemia in pregnancy is a major public health problem in sub-Saharan Africa, increasing the risk of poor maternal and newborn outcomes. Helminth infections, particularly hookworm, are a preventable but persistent cause of anemia. We studied pregnant women with intestinal helminths attending antenatal care at Fort Portal Regional Referral Hospital in western Uganda to determine how common anemia was, and what factors influenced it.

We found that about one in four helminth-infected women were anemic, with most cases being mild. Importantly, anemia risk rose with the severity of hookworm infection, showing a clear dose–response effect. Women who had recently taken deworming medication, used borehole water, or were in their second trimester were less likely to be anemic, while students also appeared protected likely reflecting better health awareness and nutrition.

These findings emphasize the importance of routine deworming, iron–folate supplementation, and safe water access during pregnancy. Strengthening antenatal care packages with timely screening, health education, and targeted interventions can reduce maternal anemia in helminth-endemic settings, ultimately improving outcomes for both mothers and babies

## Introduction

Anemia remains a significant global public health concern, affecting nearly one-quarter of the world’s population, with pregnant women among the most vulnerable groups due to increased physiological demands and risk of micronutrient deficiencies [1]. The burden is particularly high in low- and middle-income countries (LMICs), where prevalence estimates reach 57% in Sub-Saharan Africa, 48% in Southeast Asia, and 24.1% in South America [2,3]. Among the myriad of causes, helminthic infections have been increasingly recognized as significant contributors to anemia, yet remain largely neglected in maternal health interventions [4]

Intestinal helminths particularly hookworm*,* Ascaris lumbricoides, and Schistosoma mansoni are endemic in many LMICs and contribute to anemia through chronic intestinal blood loss, competition for micronutrients, and inflammatory responses [5,6]. Recent evidence indicates that the hookworm–anemia relationship in pregnancy is dose-dependent rather than merely presence-or-absence [7]. A 35-study meta-analysis found maternal anemia odds more than doubled as infection intensity increased [8]. Biologically, each adult Necator americanus or Ancylostoma duodenale consumes about 0.03 mL of host blood per day, so cumulative iron loss rises proportionally with egg-per-gram counts in stool [9]. Recognising this gradient, the World Health Organization now advises single-dose albendazole (400 mg) or mebendazole (500 mg) after the first trimester wherever moderate-to-heavy hookworm infections are common in pregnant women [10]. Evidence suggests a synergistic effect between pregnancy and helminth infections in reducing hemoglobin levels. For instance, Berhe et al. noted significantly lower hemoglobin concentrations in pregnant women co-infected with helminths [6]. Demeke et al further demonstrated that hookworm infection alone could increase the risk of anemia up to 21-fold [11]. Region-specific studies have reported a higher prevalence of anemia among helminth-infected pregnant women: 55.6% in Ethiopia versus 16.4% in uninfected counterparts [12], and 88.7% in India compared to 56.4% among those without infection [13]. However, evidence from Uganda remains limited and fragmented, with most studies focusing on helminth prevalence or the efficacy of deworming drugs rather than the direct burden and patterns of anemia [14].

The severity of anemia in helminth-infected women also varies across settings. A literature review by Mattew and Nsaidzeka reported that moderate and severe anemia were not uncommon in helminthiasis cases. Nonetheless, most studies indicate mild anemia to be most prevalent [15]. For example, Demeke et al. in Ethiopia and Azhar et al. in India both found that the majority of helminth-infected pregnant women had mild anemia, with no reported cases of severe anemia [11,13]. Similar findings were observed in Nigeria, where 25% had mild and 29% moderate anemia, but none had severe anemia [16]. In Uganda, the association between helminthiasis and anemia severity remains understudied, particularly in the western region.

Furthermore, several socio-demographic and clinical factors have been associated with anemia in pregnancy, including maternal age, education level, socioeconomic status, dietary intake, intestinal parasitic infections, and comorbidities such as malaria [16–18]. Behavioral and environmental risk factors such as geophagy, barefoot gardening, and consumption of contaminated water also contribute to both helminth infections and anemia [19,20]

Despite the high burden of anemia and the endemicity of helminthiasis in Uganda, particularly in rural western regions, there remains a critical evidence gap on the epidemiology of anemia among helminth-infected pregnant women. Notably, no prior Ugandan study has quantified the dose–response relationship between hookworm intensity and maternal anemia. The primary objective was to assess whether hookworm infection intensity is associated with maternal anemia among helminth-infected pregnant women (dose–response relationship). We hypothesized that increasing hookworm egg burden (EPG) would be associated with higher odds of anemia. Secondary objectives were to describe anemia severity distribution and to identify other independent factors associated with anemia.

## Materials and methods

### Ethics statement

This research project was approved by the research ethics committee of Kampala International University and the administration of Fort Portal Regional Referral Hospital (FRRH) under registration number KIU-REC-2022–159. The study was registered with the Uganda National Council for Science and Technology (UNCST). All study participants provided written informed consent. All ethical standards were followed according to the Declaration of Helsinki.

### Study design, population and setting

This hospital-based cross-sectional study was conducted at the antenatal clinic of Fort Portal Regional Referral Hospital (FRRH) between 1st November 2022 and 1st March 2023. FRRH, located in Fort Portal Municipality approximately 295 km west of Kampala, Uganda’s capital, has a capacity of 384 beds and serves the Tooro region, which comprises eight districts (Bundibugyo, Kabarole, Kyenjojo, Kasese, Kamwenge, Kyegegwa, Bunyangabu, and Ntoroko) as well as parts of eastern Democratic Republic of Congo. The hospital provides both general and specialized medical services and functions as a teaching site for Kampala International University and other institutions. Its modern laboratory is equipped to perform a wide range of diagnostic tests, including complete blood counts and stool analyses

### Inclusion criteria

All pregnant women diagnosed with helminthiasis who attended antenatal care (ANC) at Fort Portal Regional Referral Hospital (FRRH) during the study period were eligible for inclusion.

### Exclusion criteria

Pregnant women with helminthiasis who had taken anthelmintic treatment within the two weeks preceding recruitment, those with a history of chronic medical conditions such as sickle cell disease, chronic liver or renal disease, and those who were severely ill, mentally incapacitated, or unable to communicate were excluded from the study

### Sample size determination

The sample size was estimated using the single population proportion formula as suggested by Kish and Leslie (1965):


$$n=\frac{z^{\text{2}}\;p(\text{1}-p)}{d^{\text{2}}}$$

Where: *n* = required sample size, *Z* = 1.96 (standard normal deviate for 95% confidence level), *p* = 0.556, estimated prevalence of anemia among helminthic-infected pregnant women from a study [12], d = 0.05 (margin of error)


$$n=\frac{{\text{1}.\text{9}\text{6}}^{\text{2}}*\text{0}.\text{5}\text{5}\text{6}(\text{1}-\text{0}.\text{5}\text{5}\text{6})}{{\text{0}.\text{0}\text{5}}^{\text{2}}}=380.6$$

Thus, a total of **381 participants** were required. By adding 10% (381*0.1) of the expected non-respondents, the sample size used in this study became **420 participants**.

### Sampling technique

Participants were enrolled consecutively until the target sample of 420 was reached. This number was considered adequate, and every individual met the inclusion criteria.

### Study procedure and specimen collection

Eligible pregnant women attending ANC at Fort Portal Regional Referral Hospital (FRRH) were informed about the study and provided written consent prior to participation. Stool samples were collected and examined for helminth ova using the Kato-Katz technique. Women free of helminth infection received routine antenatal care, while those diagnosed with helminthiasis were enrolled. A structured questionnaire was administered to capture sociodemographic and clinical data. Venous blood (2–4 mL) was drawn aseptically into EDTA tubes for hemoglobin estimation using a calibrated SYSMEX XN-550 automated hematology analyzer. Anemia was defined according to WHO criteria (<11.0 g/dL in the first/third trimester and <10.5 g/dL in the second trimester). Data were recorded on standardized forms covering stool results, hemoglobin levels, and participant characteristics.

### Stool examination techniques

Stool samples were examined microscopically using the Kato-Katz thick smear technique (single slide per specimen), which is recommended by the World Health Organization for quantifying helminth egg counts. This method allowed for classification of hookworm infection intensity based on eggs per gram of stool (epg). Hookworm egg counts were categorized according to WHO morbidity thresholds: light (<2000 epg), moderate (2000–3999 epg), and heavy (≥4000 epg. Stool specimens were processed promptly on receipt. Kato-Katz thick smears were prepared and examined within 30–60 minutes of preparation to minimize under-detection due to rapid hookworm egg clearance. eggs per gram of stool (EPG)

### Study variables

Independent variables included sociodemographic, clinical, obstetric, and environmental factors, as well as knowledge about anemia in pregnancy (e.g., knowledge of causes and consequences) assessed via questionnaire. The dependent variable was anemia among pregnant women with helminthiasis as binary outcome. Hookworm intensity was categorized as light, moderate, or heavy according to WHO morbidity thresholds as described in the stool examination techniques subsection. Anemia severity was classified using WHO thresholds: mild (Hb 10.0–10.9 g/dL), moderate (Hb 7.0–9.9 g/dL), and severe (Hb < 7.0 g/dL) [21]

### Quality control

Eligibility criteria were applied consistently to ensure data accuracy. Research assistants received two days of training on study tools, consent, interviewing, and sample collection. Questionnaires were checked on-site for completeness and administered in English or Rutooro for clarity. Stool samples were transported in cool boxes (4–8 °C) and blood samples analyzed within two hours of collection. All specimens were uniquely coded to maintain confidentiality. The SYSMEX XN-550 analyser was calibrated daily with manufacturer controls (±2 SD). To ensure reliability, a senior technologist re-examined 10% of stool and blood samples, with >95% concordance (kappa >0.80), indicating substantial inter-observer agreement.

### Data management and analysis

Data from questionnaires and laboratory forms were entered into Microsoft Excel 2017 and analyzed using SPSS version 23.0. Continuous variables were summarized using means ± standard deviations (SD) or medians with interquartile ranges (IQR), as appropriate, while categorical variables were summarized using frequencies and percentages. The prevalence of anemia was calculated as the proportion of helminth-positive women with hemoglobin <11.0 g/dL in the first/third trimester or <10.5 g/dL in the second trimester. Anemia severity (mild, moderate, severe) was presented as percentages among women with anemia. Associations between explanatory variables and anemia were assessed using binary logistic regression. Hookworm infection intensity (light, moderate, and heavy) was treated as the primary exposure and included a priori in all multivariable models. Other covariates were selected based on biological plausibility and/or bivariate screening (p ≤ 0.20). Collinearity was assessed prior to fitting the final model. Results are reported as adjusted odds ratios (aORs) with 95% confidence intervals (CIs), and statistical significance was set at p < 0.05.

## Results

### Basic characteristics of the study participants

A total of 1,438 pregnant women attending antenatal care at FRRH were approached for participation. Of these, 207 failed to meet the study’s eligibility criteria and were excluded. The remaining 1,231 women provided stool samples, and 420 (34.1%) tested positive for helminthiasis and were enrolled. The final analytic sample therefore comprised 420 participants.

The majority of the respondents were aged 20–29 years (275, 65.5%), resided in urban areas (222, 52.9%), were married (370, 88.1%), and the largest proportion had attained primary education (174, 41.4%). Most husbands had completed secondary schooling (252, 60%). Business was the leading occupation (136, 32.4%), followed by housework (98, 23.3%) and subsistence farming (85, 20.2%). Over half lived in households of two to four members (227, 54%). Nearly all identified as Christian (386, 91.9%) and relied chiefly on tap water (196, 46.7%) or boreholes (193, 46.0%) for drinking (Table 1).

**Table 1:** Descriptive statistics for socio-demographic characteristics of the participants (N = 420).

Variables	Categories	Frequency(n)	Percentage (%)
Age	<20	54	12.9
	**20-29**	**275**	**65.5**
	30-39	83	19.8
	>=40	8	1.9
Residence	Rural	198	47.1
	**Urban**	**222**	**52.9**
Marital status	Single	43	10.2
	Divorced	7	1.7
	**Married**	**370**	**88.1**
Husband education level	None	38	9
	Primary	130	31
	**Secondary**	**252**	**60**
Maternal Education level	None	42	10
	**Primary**	**174**	**41.4**
	Secondary	146	34.8
	Tertiary	58	13.8
Occupation	Housewife	98	23.3
	subsistence farming	85	20.2
	Student	36	8.6
	**Business**	**136**	**32.4**
	Civil servant	65	15.5
Family size	5 to 10 members	193	46
	**2 to 4 members**	**227**	**54**
Source of Drinking water	Borehole	193	46
	Spring/Well	31	7.4
	**Tap water**	**196**	**46.7**
Religion	Muslim	34	8.1
	**Christian**	**386**	**91.9**

### Prevalence and severity patterns of anemia among helminth-infected pregnant women attending ANC at FRRH (N=420)

**Fig 1:**
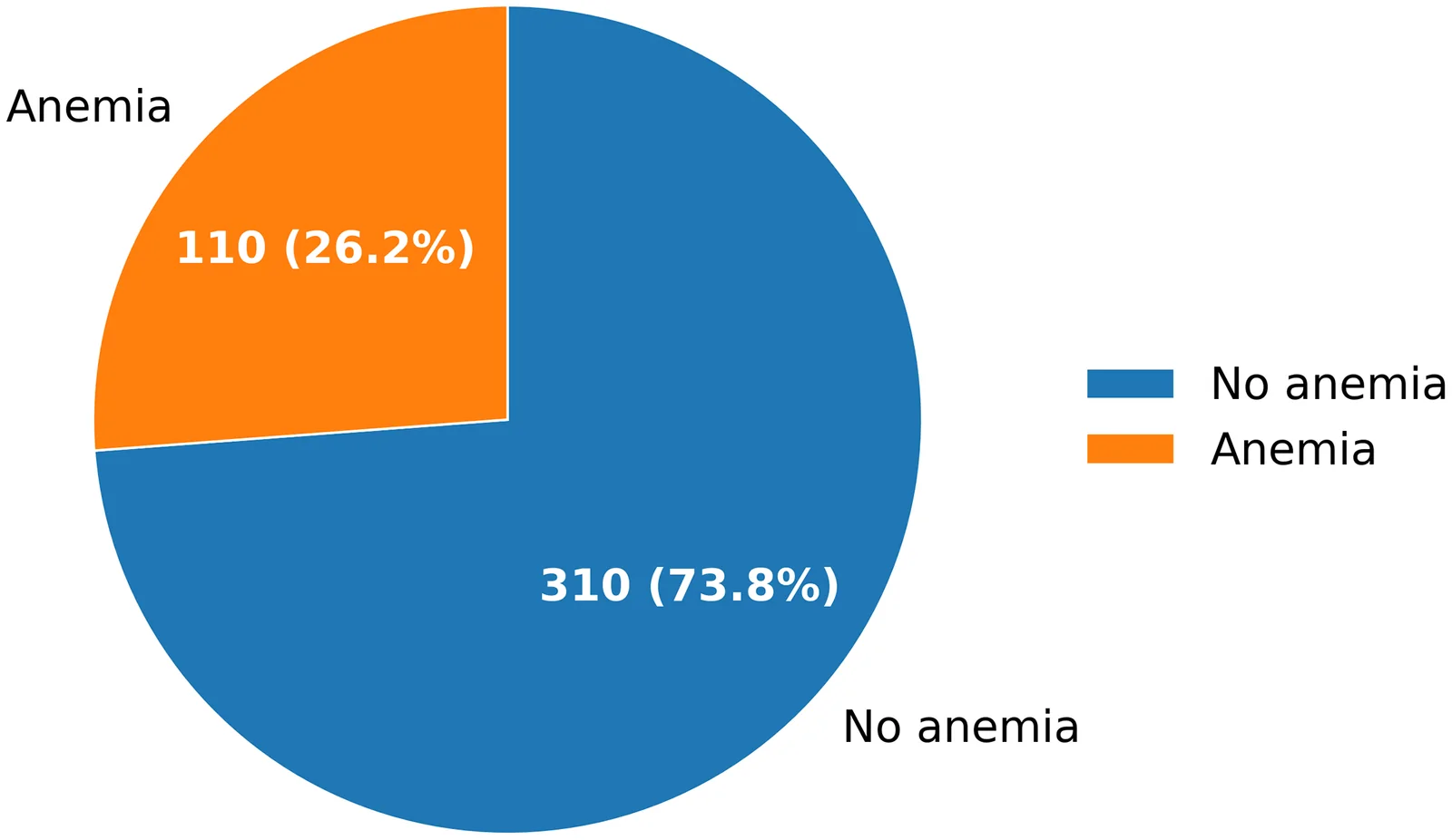
Pie chart showing the prevalence of anemia among pregnant women with helminthiasis attending ANC at FRRH.

**Fig 2:**
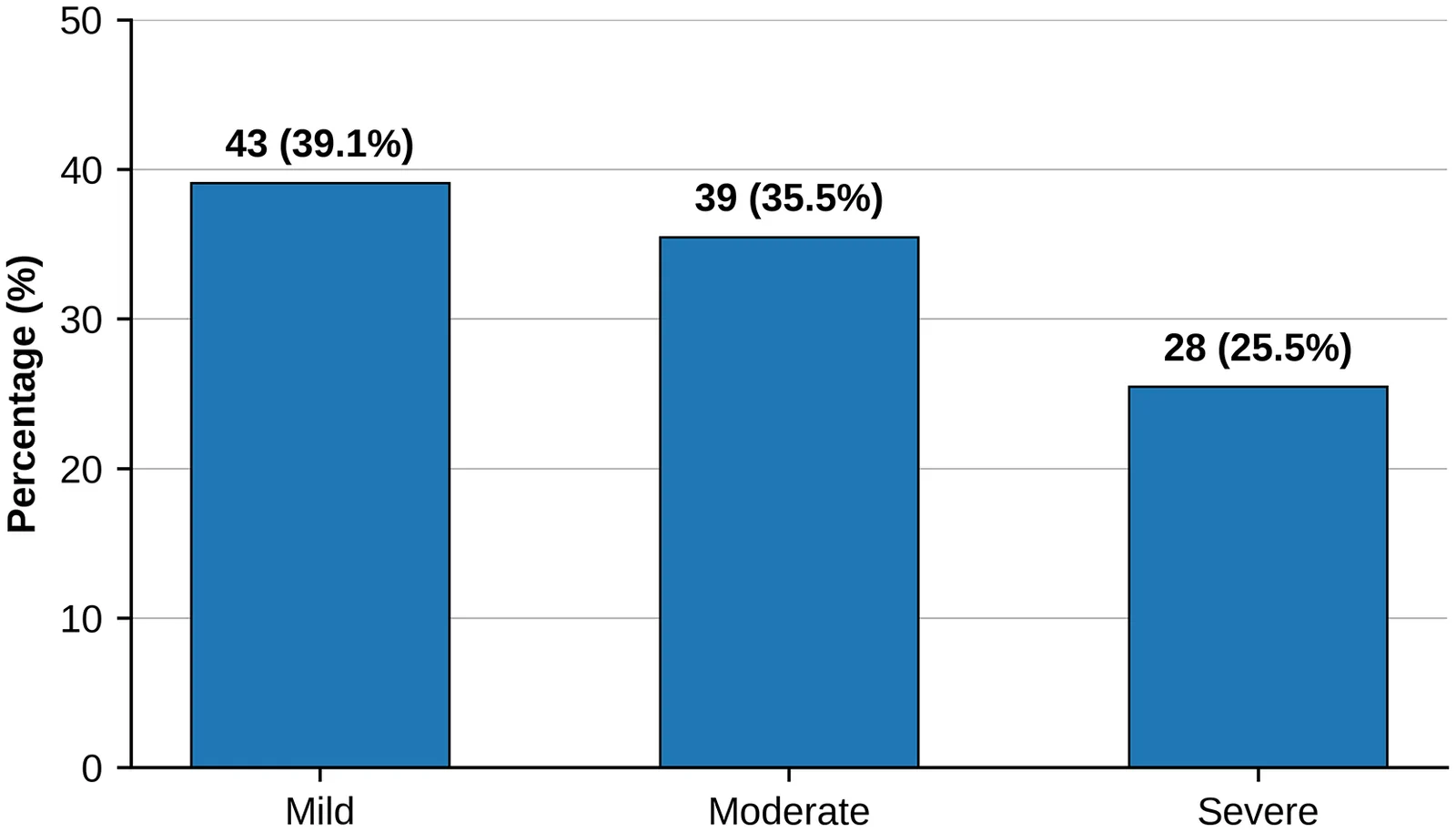
A pie chart representing the severity patterns of anemia among helminth-infected pregnant women.

Of the 420 helminth-positive pregnant women included in the study at FRRH, 110 (26.2%) were anemic, while 310 (73.8%) had normal hemoglobin (Fig 1). Among the 110 anemic participants, 43 (39.1%, 95% CI: 30.5–48.4) had mild anemia, 39 (35.5%, 95% CI: 27.1–44.7) had moderate anemia, and 28 (25.5%, 95% CI: 18.2–34.3) had severe anemia (Fig 2).

### Factors associated with anemia among helminth-infected pregnant women (N=420)

The bivariate analysis showed that hookworm intensity, rural residence, husbands with no formal education, student status, business occupation, recognizing bleeding as a cause of anemia, having received an anthelminthic dose ≥3 months earlier, use of borehole or spring/well water, consuming one or two meals daily, a history of urinary tract infection or malaria during the current pregnancy, gestational age 14–27 weeks, and attending one to four antenatal visits were associated with anemia among helminth-infected pregnant women (Tables 2,3).

**Table 2:** Bivariate analysis of the association between hookworm intensity and anemia among hookworm-infected pregnant women attending ANC at FRRH (N = 275).

Variables	Categories	Anemia		cOR [95% - CI]	p
		No (198)	Yes (77)		
Hookworm Intensity	Light	108 (79.4)	28 (20.6)	Ref	
	Moderate	74 (68.5)	34 (31.5)	1.82 (1.02–3.22)	0.042*
	Heavy	16 (51.6)	15 (48.4)	3.64 (1.55–8.51)	0.004*

cOR = Crude Odds Ratio, CI = Confidence Interval, p = Significance (<0.2), Ref = Reference

**Table 3:** Bivariate analysis of factors associated with anemia among helminth-infected pregnant women (N = 420).

Variables	Categories	Anemia		cOR [95% - CI]	p
		No (310)	Yes (110)		
Age	<20	40(74.1)	14(25.9)	2.45(0.276-21.715)	0.421
	20-29	205(74.5)	71(25.5)	2.424(0.293-20.050)	0.411
	30-39	58(69.9)	25(30.1)	3.017(0.352-25.828)	0.313
	≥40	7(87.5)	1(12.5)	Ref	
Residence	Rural	152(76.8)	46(23.2)	0.747(0.481-1.159)	0.194*
	Urban	158(71.2)	64(28.8)	Ref	
Marital status	Single	32(74.4)	11(25.6)	0.967(0.469-1.994)	0.929
	Divorced	5(71.4)	2(28.6)	1.126(0.215-5.898)	0.888
	Married	273(73.8)	97(26.2)	Ref	
Husband education level	None	24(63.2)	14(36.8)	1.644(0.803-3.365)	0.174*
	Primary	100(76.9)	30(23.1)	0.846(0.515-1.387)	0.507
	secondary	186(73.8)	66(26.2)	Ref	
Maternal Education level	None	26(61.9)	16(38.10	1.368(0.593-3.152)	0.463
	Primary	133(76.4)	41(23.6)	0.685(0.355-1.322)	0.259
	secondary	111(76.0)	35(24.0)	0.701(0.357-1.374)	0.301
	Tertiary	40(69.0)	18(31.0)	Ref	
Occupation	Housewife	72(73.5)	26(26.5)	0.659(0.335-1.299)	0.229
	Subsistence farming	61(71.8)	24(28.2)	0.718(0.359-1.438)	0.35
	Student	33(91.7)	3(8.3)	0.166(0.046-0.601)	0.006*
	Business	102(75.0)	34(25.0)	0.609(0.321-1.154)	0.128*
	Civil servant	42(64.6)	23(35.4)	Ref	
Family size	5 to 10 members	147(76.2)	46(23.8)	0.797(0.513-1.237)	0.312
	2 to 4 members	163(71.8)	64(28.2)	Ref	
Source of Drinking water	Borehole	151(78.2)	42(21.8)	0.713(0.449-1.132)	0.449
	Spring/Well	18(58.1)	13(41.9)	1.852(0.850-4.033)	0.85
	Tap water	141(71.9)	55(28.1)	Ref	
Religion	Muslim	28(82.4)	6(17.6)	0.581(0.234-1.443)	0.242
	Christian	282(73.1)	104(26.9)	Ref	
Causes of anemia in pregnancy	Don't know	15(75.0)	5(25.0)	0.833(0.292-2.375)	0.733
	Bleeding	106(79.1)	28(20.9)	0.66(0.402-1.085)	0.101*
	Infection	4(57.1)	3(42.9)	1.875(0.410-8.582)	0.418
	Under nutrition	185(71.4)	74(28.6)	Ref	
Consequences of anemia in pregnancy	Don't know	20(66.7)	10(33.3)	1.425(0.631-3.216)	0.394
	Low birth weight	31(75.6)	10(24.4)	0.919(0.425-1.988)	0.831
	Poor baby growth	34(72.3)	13(27.7)	1.09(0.539-2.202)	0.811
	Baby death	54(76.1)	17(23.9)	0.897(0.483-1.667)	0.731
	Maternal death	171(74.0)	60(26.0)	Ref	
Whether given dewormer	No	57(77.0)	17(23.0)	0.811(0.449-1.466)	0.488
	Yes	253(73.1)	93(26.9)	Ref	
Last time of Helminthic meds	Never taken	60(74.1)	21(25.9)	0.947(0.543-1.651)	0.848
	Don't remember	7(70.0)	3(30.0)	1.16(0.293-4.587)	0.833
	≥3months	13(92.9)	1(7.1)	0.208(0.027-1.615)	0.133*
	<3months	230(73.0)	85(27.0)	Ref	
Source of Drinking water	Borehole	151(78.2)	42(21.8)	0.713(0.449-1.132)	0.152*
	Spring/Well	18(58.1)	13(41.9)	1.852(0.850-4.033)	0.121*
	Tap water	141(71.9)	55(28.1)	Ref	
Number of meals per day	1 meal	3(37.5)	5(62.5)	5.264(1.231-22.504)	0.025*
	2 meals	48(67.6)	23(32.4)	1.513(0.868-2.638)	0.144*
	>2 meals	259(76.0)	82(24.0)	Ref	
Medical conditions ever had	Malaria	95(71.4)	38(28.6)	1.187(0.678-2.076)	0.549
	UTI+ Malaria	3(50.0)	3(50.0)	2.967(0.568-15.493)	0.197*
	UTI	123(75.9)	39(24.1)	0.941(0.543-1.628)	0.827
	None	89(74.8)	30(25.20	Ref	
Gravidity	Primigravida	77(72.6)	29(27.4)	1.083(0.66-1.779)	0.752
	Multigravida	233(74.2)	81(25.8)	Ref	
Gestational Age	<14w	4(66.7)	2(33.3)	1.089(0.194-6.103)	0.923
	14-27w	171(78.8)	46(21.2)	0.586(0.376-0.912)	0.018*
	≥28w	135(68.5)	62(31.5)	Ref	
Number of ANC visits	Once	58(77.3)	17(22.7)	0.573(0.274-1.199)	0.14*
	2–4	207(74.7)	70(25.3)	0.662(0.374-1.171)	0.156*
	≥4	45(66.2)	23(33.8)	Ref	

cOR = Crude Odds Ratio, CI = Confidence Interval, p* = Significance (<0.2), w = week, Ref = Reference, UTI = Urinary Tract Infection

In the multivariable model, after adjustment for potential confounders (p < 0.05), five factors remained independently associated with anemia (Table 4). Compared with light hookworm intensity, moderate and heavy hookworm intensity were associated with higher odds of anemia (aOR = 1.902, 95% CI: 1.164–3.543; p = 0.028 and aOR = 2.400, 95% CI: 1.063–5.409; p = 0.041, respectively). In contrast, compared with their respective reference groups, being a student, having last received anthelminthic treatment more than 3 months earlier, relying on borehole water, and being in the 14–27 weeks’ gestational age group were associated with lower odds of anemia (aOR = 0.167, 95% CI: 0.045–0.627; p = 0.008; aOR = 0.107, 95% CI: 0.013–0.882; p = 0.038; aOR = 0.598, 95% CI: 0.359–0.995; p = 0.048; and aOR = 0.567, 95% CI: 0.357–0.915; p = 0.020, respectively).

**Table 4:** Multivariable logistic regression of factors associated with anemia among helminth-infected pregnant women (N = 420).

Variables	Categories	cOR (95% - CI)	*p*	aOR (95% - CI)	*P*
Hookworm intensity	Light	Ref		Ref	
	Moderate	1.823 (1·015–3·221)	0.042	1.902(1.164–3.543)	0.028 **
	Heavy	3.642 (1·552–8·512)	0.004	2.412(1·063–5·409)	0.041 **
Occupation	Housewife	0.659(0.335-1.299)	0.229	0.67(0.324-1.387)	0.281
	Subsistence farming	0.718(0.359-1.438)	0.35	0.848(0.393-1.831)	0.675
	Student	0.166(0.046-0.601)	0.006	0.167(0.045-0.627)	0.008 **
	Business	0.609(0.321-1.154)	0.128	0.635(0.329-1.228)	0.177
	Civil servant	Ref		Ref	
Last time of Helminthic meds	Never taken	0.947(0.543-1.651)	0.848	1.184(0.632-2.219)	0.598
	Don't remember	1.16(0.293-4.587)	0.833	1.569(0.375-6.573)	0.538
	≥3months	0.208(0.027-1.615)	0.133	0.107(0.013-0.882)	0.038 **
	<3months	Ref		Ref	
Source of Drinking water	Borehole	0.713(0.449-1.132)	0.152	0.598(0.359-0.995)	0.048 **
	Spring/Well	1.852(0.850-4.033)	0.121	2.298(0.949-5.563)	0.065
	Tap water	Ref		Ref	
Gestational Age	<14w	1.089(0.194-6.103)	0.923	1.093(0.175-6.823)	0.924
	14-27w	0.586(0.376-0.912)	0.018	0.567(0.351-0.915)	0.020 **
	≥28w	Ref		Ref	

cOR = Crude Odds Ratio, CI = Confidence Interval, aOR = Adjusted Odds Ratio, p** = Significance, w = week.

## Discussion

We found a 26.2% prevalence of anemia among helminth-infected pregnant women, with a clear dose–response relationship between hookworm intensity and anemia risk. This prevalence is substantially lower than the 55.6% reported in Ethiopia [12], but higher than the 17.5% reported by Dorsamy et al. in Ethiopia [1]. Differences in sample size, altitude, dietary iron intake, and the timing or uptake of preventive chemotherapy may explain this variability. Other studies have documented higher prevalence rates: 39.7% in Entebbe, Uganda [22], 47.3% in Peru [23], and 58% in Nepal [24], reflecting heavier helminth burdens or weaker deworming coverage. Conversely, systematic mass-drug-administration programs, as seen in Ethiopia [1], demonstrate that sustained deworming can meaningfully curb helminth-related anemia. A Nigerian rural cohort reported 40% [25], suggesting co-endemic malaria, dietary limitations, and poor supplementation further compound risk. These findings highlight the heterogeneity of helminth-related anemia across settings and emphasize the influence of local context, including nutritional practices, deworming coverage, malaria co-infection, gestational age, and methodological differences in defining anemia.

With respect to severity, we observed 39.1% mild, 35.5% moderate, and 25.5% severe anemia among anemic participants. Mild anemia predominated, consistent with studies from Ethiopia [11] and India [13], although absolute distributions varied. By contrast, a Nigerian cohort recorded more moderate anemia and few severe cases despite higher overall prevalence [16]. These discrepancies may reflect differences in gestational age at recruitment, infection intensity, supplementation practices, or health service delivery. Collectively, evidence suggests that while mild anemia is the most common manifestation, the proportion of moderate and severe cases increases where hookworm burdens are high and nutritional support is limited.

In the multivariable analysis, moderate and heavy hookworm intensity were independently associated with nearly two-fold and 2.4-fold higher odds of anemia, respectively. This graded association is consistent with a global meta-analysis showing that the risk of anemia increases more than two-fold with increasing worm burden [8]. The biological plausibility is strong: each adult Necator americanus or Ancylostoma duodenale consumes 0.03 mL of blood per day, leading to cumulative iron loss proportional to egg-per-gram counts [9]. This underscores that infection intensity, not mere presence, is the critical determinant of maternal hemoglobin

Several factors were protective against anemia in this population. Student status conferred 80% reduced odds, echoing Ethiopian data linking students to lower odds of anemia [26]. The lower odds observed among students should also be interpreted relative to civil servants rather than as an absolute ranking of socioeconomic position. Student status may be linked to higher health literacy and greater exposure to nutrition/ANC counseling, which can improve adherence to iron–folic acid supplementation and dietary practices [27,28]. Compared with women who reported anthelminthic treatment within the past three months, those whose last treatment was three months or more prior had lower odds of anemia. Evidence from antenatal deworming trials suggests that hemoglobin improvements may emerge later in pregnancy, which supports the plausibility that treatment received earlier could be associated with lower anemia at the time of assessment [29]. This seemingly counterintuitive finding may also reflect confounding by indication (e.g., women with symptoms or lower hemoglobin being more likely to receive recent treatment) and the inability of a cross-sectional design to establish temporality [30]. Use of borehole water was protective, likely reflecting lower exposure to contaminated sources and supporting reviews linking improved water sources to reduced helminth infection [19]. Finally, being in the second trimester (14–27 weeks) reduced anemia risk by 43%, similar to reports that anemia often peaks outside mid-pregnancy due to hemodilution and national deworming program schedules [19]. Grouped together, these findings reinforce that both socio-behavioral and environmental factors influence anemia outcomes in helminth-endemic settings.

From a policy perspective, our findings support World Health Organization recommendations for routine single-dose albendazole (400 mg) or mebendazole (500 mg) after the first trimester in areas with moderate-to-heavy hookworm infections (WHO, 2023). Notably, 35% of our participants carried ≥2000 EPG, underscoring the need to integrate systematic stool screening, timely anthelminthic treatment, and iron–folate supplementation into Uganda’s antenatal-care package. Evidence from Uganda [14] and a Cochrane meta-analysis [31] shows that such integration can substantially reduce maternal anemia. Strengthening water access, nutrition education, and community deworming programs could yield additional gains.

### Study strengths and limitations

This is the first documented study at Fort Portal Regional Referral Hospital to quantify the prevalence of, and factors associated with, anemia among pregnant women with helminthiasis. Residual confounding is possible because some contributors to anemia in pregnancy (e.g., dietary iron intake, micronutrient status, and other comorbidities) may not have been fully measured. Because this was a hospital-based study of antenatal attendees, the observed prevalence may not reflect the true community burden, limiting generalizability to all pregnant women, including those who do not attend ANC or deliver outside facilities. In addition, the cross-sectional design precludes causal inference and clear temporality between exposures and anemia. Future population-based, longitudinal studies are needed to validate these findings and clarify temporal relationships

## Conclusions and recommendations

This study found a relatively lower prevalence of anemia among helminth-infected pregnant women at Fort Portal Regional Referral Hospital compared with regional reports with mild anemia predominating. A clear dose–response effect was observed, as moderate and heavy hookworm intensities significantly increased anemia risk. In contrast, student status, prior deworming ≥3 months, borehole water use, and second-trimester gestation were independently protective. These findings highlight persistent gaps in routine deworming, iron–folate supplementation, safe-water access, and nutrition support, underscoring the need to strengthen antenatal care through systematic stool screening, timely provision of anthelminthic therapy, improved supplementation and counselling, promotion of safe water sources, and targeted health education. Tailoring antenatal care by anemia severity and prioritizing high-burden women are also essential to reduce the impact of maternal anemia in helminth-endemic settings.

## Supporting information

S1 Data De-identified dataset underlying the findings reported in this study. (XLSX)
